# AL3810, a multi-tyrosine kinase inhibitor, exhibits potent anti-angiogenic and anti-tumour activity *via* targeting VEGFR, FGFR and PDGFR

**DOI:** 10.1111/j.1582-4934.2012.01541.x

**Published:** 2012-09-26

**Authors:** Yuanfeng Zhou, Yi Chen, Linjiang Tong, Hua Xie, Weiwei Wen, Jie Zhang, Yong Xi, Yanyan Shen, Meiyu Geng, Yuanyuan Wang, Hualiang Jiang, Cheng Luo, Liping Lin, Jian Ding

**Affiliations:** a Division of Anti-Tumor Pharmacology State Key Laboratory of Drug Research, Shanghai Institute of Materia Medica Chinese Academy of ScienceShanghai, China; b Drug Discover and Design Center State Key Laboratory of Drug Research, Shanghai Institute of Materia Medica Chinese Academy of ScienceShanghai, China

**Keywords:** multi-tyrosine kinases inhibitor, angiogenesis, anti-tumour activity

## Abstract

Angiogenesis plays an important role in neoplastic transformation and progression as well as in the metastasis process of most human cancers. Herein, we identified AL3810 as a novel and orally bioavailable small molecular inhibitor with potent inhibitory activity against multiple tyrosine kinases involved in the process of angiogenesis. We found that AL3810 substantially inhibited the autophosphorylation of VEGFR2, PDGFRβ and FGFR1 in endothelial cells. Moreover, AL3810 exhibited potent anti-angiogenesis activity, manifested by significant inhibition of microvessel outgrowth of rat arterial ring and chickallantochorion membrane (CAM) in *ex vivo* angiogenesis models. Daily dosing of AL3810 has shown broad-spectrum anti-tumour activity in human kidney, pancreas, liver cancer xenograft models. Importantly, immunohistochemistry results demonstrated that the anti-tumour activity of AL3810 was closely correlated with its anti-angiogenesis activity, as demonstrated by a decreased microvessel area and reduced microvessel numbers in tumour tissues. The overall pharmacological profiles of AL3810 are superior to sorafenib. The clinical trials of AL3810 will soon be launched in China.

## Introduction

Angiogenesis, the growth of new vessels from pre-existing vasculature within a tumour tissue, is important in tumour progression involved in both local tumour growth and development of distant tumour metastases [1]. It has been validated that multiple tyrosine kinases are involved in angiogenesis [2]. VEGF signalling through its receptor, in particular VEGFR2, is a major inducer in this setting. This pathway activates downstream network [3], and dominantly stimulates endothelial cells proliferation and migration and subsequent organization of tube formation [4–7]. Whereas the pivotal role of VEGF and VEGFR-2 has been amply documented, one particular line of evidence, based on pre-clinical animal models, suggests that targeting VEGF-VEGFR signalling and focusing on endothelial cells is beneficial at the start of treatment, but with continued drug treatment and under the pressure of VEGF signalling blockade resulting in increased hypoxia and malnutrition on the tumour cells, other signalling molecules and their cognate receptors provide alternate mechanisms to drive disease progression. Among the potential compensatory mechanisms, the platelet-derived growth factor (PDGF) and fibroblast growth factor (FGF) pathways have been identified as promising targets for optimized drug candidates. The PDGF receptor (PDGFR) functions *via* binding to two different receptors, known as PDGFR-α and-β [8], which facilitates the recruitment of pericytes and smooth muscle cells, and is important for maturation and stability of the vasculature [9]. Studies have demonstrated that interference with PDGFBB/ PDGFRβ signalling resulted in disruption of already established endothelial/pericyte associations and vessel destabilization during retinal development [10]. Therefore, the use of a PDGFRβ tyrosine kinase inhibitor (TKI) should provide a novel strategy to interfere with pericyte function during tumour angiogenesis [11]. The basic FGF (b-FGF)/FGF receptor (FGFR) axis is another pathway associated with angiogenesis. FGFs were the first identified angiogenic factors and their roles in endothelial cell proliferation, migration, cell adhesion and other angiogenic promoting processes have been extensively studied [12]. Moreover, there is ample evidence suggesting that FGF induce the expression of VEGF in vascular endothelial cells, while blocking antibodies against VEGF [13, 14].

Increasing understanding of the molecular mechanisms that regulate angiogenesis has intrigued an appeal to developing anti-angiogenic tyrosine kinases as a systemic strategy for cancer treatment [15]. Tremendous efforts have been invested in the discovery of such inhibitors, and three distinct anti-angiogenic tyrosine kinase inhibitors, sunitinib, sorafenib and pazopanib, with differential targeting capacities to angiogenic kinases, have been approved for the treatment of patients with advanced cancer (renal cell cancer, gastro-intestinal stromal tumours and hepatocellular cancer).

Given the complexity of tumour angiogenesis, simultaneously blocking FGF and PDGF signalling pathways during the inhibition of VEGF pathway may maximize the anti-tumour efficacy and minimize the acquired resistance to VEGF-targeted therapy. In addition, the structural similarities between VEGFR, PDGFR and FGFR tyrosine kinases provide a window for medicinal chemistry to design inhibitors that are active on all three kinase families [16]. In this study, using combinatorial lead-optimization strategy we synthesized 6-[[7-[(1-aminocyclopropyl)methoxy]-6-methoxy-4-quinolyl]oxy]-*N*-methyl-naphthalene-1-carboxamide, designated as AL3810, also named E-3810 [17], which is systematically identified as a novel, orally bioavailable small molecular inhibitor targeting multiple angiogenic kinases, includingVEGFR1, VEGFR2, FGFR1 and PDGFRβ, with an IC_50_ at low nanomolar level. We further demonstrated that this compound exhibits potent anti-angiogenesis activity and significantly inhibits the tumour growth in a broad range of established human cancer xenografts models. The immunohistochemistry analysis revealed that the anti-tumour activity of AL3810 correlates well with its anti-angiogenic potency. All these provide AL3810 as a promising multi-targeted angiogenic inhibitor, deserving further development.

## Material and methods

### Compounds

**Fig 1:**
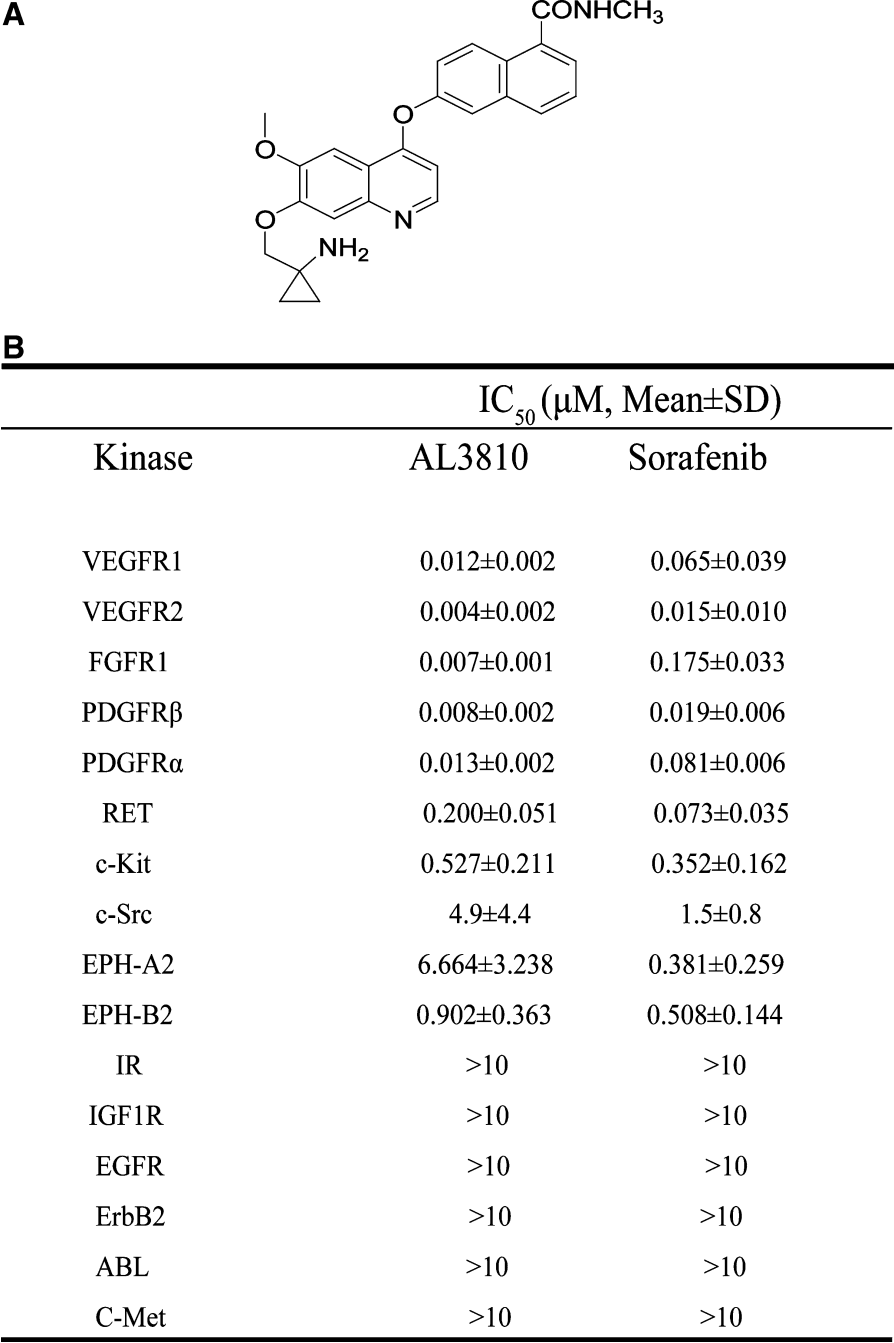
Effects of AL3810 on the activity of a panel of tyrosine kinases. (A) The chemical structure of AL3810. (B) Inhibitory activity of AL3810 against 16 different tyrosine kinases. Kinase activity was assayed by ELISA, concentrations that cause 50% inhibition (IC_50_) are shown as Mean ± S.D. of three to six separate experiments performed in duplicate.

AL3810 (chemical structure shown in Fig. 1) is originally invented by US Advenchen labs. Sorafenib was synthesized by ChemPartner Co. Ltd (Hangzhou, China). In *in vitro* studies, these compounds were dissolved to 10mM with DMSO as a stock solution and stored at −20°C. In *in vivo* studies, AL3810 was dissolved with 0.5% carboxymethyl cellulose sodium and sorafenib was prepared as reference [18].

### Cell lines

Human umbilical vein endothelial cells (HUVECs) were isolated as previously described [19], and presence of von Willebrand factor was confirmed by immunofluorescence staining. HUVECs were cultured in M199 medium supplemented with 20% heat-inactivated foetal bovine serum (FBS) containing 30 mg/ml endothelial cell growth supplement, 10 ng/ml epidermal growth factor, 100 units/ml penicillin and 100 units/ml streptomycin. Cells at passages 3–5 were used for experiments. The hepatocellular carcinoma BEL-7402, SMMC-7721 cell lines were obtained from Shanghai Institute of Materia Medica, Chinese Academy of Sciences, pancreatic cancer BXPC-3, renal cell carcinoma 786-O and Swiss3T3 cell lines were purchased from the American Type Culture Collection (Manassas, VA, USA). All cells, except Swiss3T3 were maintained in RPMI 1640 supplemented with 10% heat-inactivated foetal bovine serum. Swiss3T3 and NIH3T3-VEGFR2 cells were cultured with DMEM supplemented with 10% heat-inactivated foetal bovine serum. NIH3T3-VEGFR2 cell was selected with 50 μg/ml G418.

### ELISA kinase assay

The kinase domain of VEGFR2,c-KIT,c-Src, IGF1R, IR, EPHA2, EPHB2, EGFR, ErbB2 and c-Met were expressed using the Bac-to-Bac™ baculovirus expression system (Invitrogen, Carlsbad, CA, USA) and purified on Ni-NTA columns (QIAGEN Inc., Valencia, CA, USA) as previously described [20]. Recombinant FGFR1, RET, PDGFRα, PDGFRβ, VEGFR1 and ABL proteins were obtained from Upstate Biotechnology. The tyrosine kinase activities of the purified VEGFR2, c-KIT,c-Src, IGF1R, IR, EPHA2, EPHB2, EGFR, ErbB2 and c-Met kinase domains were determined using ELISA methodology described previously [21]. Briefly, 50 μl 10 μM ATP solution diluted in kinase reaction buffer (50 mM HEPES pH 7.4, 20 mM MgCl_2_, 0.1 mM MnCl_2_, 0.2 mM Na_3_VO_4_, 1 mM DTT) was added to each well. Various concentrations of compounds diluted in DMSO were added to each reaction well. A total volume of 10 μl DMSO was used as the vehicle control. The kinase reaction was performed in triplicate and initiated by adding purified tyrosine kinase proteins diluted in 40 μl of kinase reaction buffer. After incubation for 60 min. at 37°C, the plate was washed three times with PBS containing 0.1% Tween 20 (T-PBS). Next, 100 μl anti-phosphotyrosine (PY99) antibody (1:500; Santa Cruz Biotechnology, Santa Cruz, CA, USA) diluted in T-PBS containing 5 mg/ml BSA was added and the plate was placed at 37°C for 30 min. After the plate was washed three times, 100 μl horseradish peroxidase-conjugated goat antimouse IgG (1:2000; Calbiochem, San Diego, CA, USA) diluted in T-PBS containing 5 mg/ml BSA was added, and the plate was re-incubated at 37°C for 30 min. The plate was washed, then 100 μl citrate buffer (0.1 M, pH 5.5) containing 0.03% H_2_O_2_ and 2 mg/ml o-phenylenediamine was added and samples were incubated at room temperature until colour emerged. The reaction was terminated by adding 50 μl of 2 M H_2_SO_4_, and the plate was read using a multi-well spectrophotometer (VERSAmax™; Molecular Devices, Sunnyvale, CA, USA) at 492 nm. The inhibition rate (%) was calculated with the formula: [1−(A_492_ test/A_492 control_)] × 100%. IC_50_ values were calculated from the inhibitory curves. For the ATP competitive assay, the conditions were the same as above except the varying concentrations of ATP were added in the absence or presence of a single concentration of AL3810.

### Reversible assay

Rapid dilution experiment was used to demonstrate reversible binding of AL3810 to VEGFR2. VEGFR2 kinase (250 nM) was pre-incubated with excessive AL3810 (250 nM, a concentration of 50-fold IC_50_ for VEGFR2) under room temperature for 30 min., and then were diluted with reaction solution containing substrate peptide (5-FAM-EEPLYWSFPAKKK-CONH2) and 15 μM ATP to a hundred times, and use Caliper EZ Reader to assay the enzyme activity of this mixture. Wells were repeatedly read for 90 min. For cellular VEGFR2 autophosphorylation reversible assay, HUVEC cells were grown to half confluence in six-well plates, exposed to 1 μmol/l AL3810 for 4 hrs, then drugs were wash off with PBS, and cells were cultured with fresh medium continuatively. At different times (0, 3, 6, 24 hrs), cells were collected and the lysates were subjected to western blot.

### Western blot analysis

Cells were grown to half confluence in six-well plates, starved in serum-free medium with 1% FBS for 24 hrs, and then exposed to various concentrations of compounds for 2 hrs. For analysis of receptor tyrosine kinase phosphorylation and downstream signal transduction pathways, cells were stimulated with 50 ng/mlVEGF, 25 ng/mlb-FGF, 40 ng/ml PDGF-BB (R&D Systems, Minneapolis, MN, USA) for 10 min. at 37°C at the end of compounds treatment. Cells were then washed with cold PBS and lysed. Lysates were then subjected to SDS-PAGE, transferred to nitrocellulose membranes, blocked with 5% milk-TBST and probed with antibodies against phospho-VEGFR2 (Tyr996), phospho-VEGFR2 (Tyr1175), VEGFR2, phospho-FGFR (Tyr653/654), FGFR1, phospho-PDGFRβ (Tyr751), phospho-ERK1/2, ERK1/2, phosphor-AKT (Ser473) and AKT (Cell Signaling Technology, Beverly, MA, USA), PDGFRβ (Santa Cruz Biotechnology).

### Growth factor-induced cell proliferation assay

Cells were seeded in 96-well tissue culture plates. Next day, cells will incubated in serum free medium with 1% FBS for 24 hrs. Cells were then exposed to various concentrations of compounds for 2 hrs and then growth factor (VEGF_165_ 50 ng/ml, b-FGF 25 ng/ml, PDGF-BB 40 ng/ml) were added. Cells were cultured for another 72 hrs. Cell proliferation assay was determined using sulforhodamine B (SRB; Sigma-Aldrich, St. Louis, MO, USA).

### Molecular docking simulation

Firstly, the structures of AL3810 and sorafenib were minimized using the Tripos force field and Gasteiger-Hückel charge with distance dependent dielectric and conjugate gradient method with convergence criterion of 0.01 kcal/mol. The crystal structure of active and inactive VEGFR2 complexes with inhibitor was retrieved from Brookhaven Protein Data Bank [22] (PDB entry: 3CJG and 3EFL). All ligands and waters were removed and all hydrogen atoms were added. After the structures of receptors and ligands were carefully prepared, the Glide prediction was performed with Maestro v7.5 (Schrodinger, Inc., Portland, OR, USA) [23] in extra-precision mode, with up to 10 poses saved per molecule, for its good performance on kinase docking. Hydrogen atoms and charges were added during a brief relaxation performed with the Protein Preparation module in Maestro with the ‘preparation and refinement’ option, and a restrained partial minimization was terminated when the root-mean-square deviation (rmsd) reached a maximum value of 0.3 A to relieve steri-clashes. Finally, the interactions between the ligands and receptors were carefully analysed by LIGPLOT [24] or by pymol software [25, 26].

### Chicken chorioallantoic membrane assay (CAM)

Groups of 10 fertilized chicken eggs were transferred to an egg incubator (Lyon, Chula Vista, CA, USA) maintained at 37°C and 50% humidity and allowed to grow for 7 days. Gentle suction was applied at the hole located at the broad end of the egg to create a false air sac directly over the chicken chorioallantoic membrane, and a 1cm^2^ window was removed from the egg shell immediately. Glass coverslips (0.5 × 0.5 cm) saturated with compounds or normal saline was placed on areas between pre-existing vessels and the embryos were further incubated for 48 hrs [27]. The neovascular zones beside were photographed under a stereomicroscope (MS5; Leica, Heerbrugg, Switzerland).

### Rat aortic ring assay

The aortas were harvested from 6-week-old Sprague–Dawley rats. Each aorta was cut into 1mm slices and embedded in 90 μl Matrigel in 96-well plates. The aortic rings were fed with 100 μl of M199 with different concentrations of AL3810, and photographed on day 6 [28]. The quantity of microvessels is valued by relative area covered with microvessels using Image-Pro Express.

### Tumour xenograft experiments

Nude mice were used for all *in vivo* studies. The mice were housed and maintained under specific-pathogen free conditions in accordance with Institutional Animal Care and Use Committee. Xenografts 786-O, SMMC-7721, BEL-7402, Bxpc-3 were tumour cell lines derived, and the xenografts No.213847 and No.20090223 were fresh tumour tissue acquired from renal carcinoma patients with the approval of Ethics Committee of RenJi hospital in Shanghai. Tumour cells (5 × 10^6^) or fresh tumour tissue from patient were s.c. injected into right flank of nude mice, the tumours formed in mice were passed in nude mice to make tumours stable, and then well-developed tumours (2–3 passage) were cut into 1 mm^3^ fragments and transplanted s.c. into the right flank of nude mice using a trocar. When the tumour reached a volume 100–200 mm^3^, the mice were randomly assigned into control and treatment groups, six mice per group. Control groups were given vehicle alone, and treatment groups received AL3810 or sorafenib (p.o.). Compounds were administrated 5 days per week for the duration indicated in each treatment. The sizes of the tumours were measured twice per week using microcalipers. The tumour volume (*V*) was calculated as follows: V = (length × width^2^) / 2. The individual relative tumour volume (RTV) was calculated as follows: RTV = *V*_t_/*V*_0_, where *V*_t_ is the volume on each day, and *V*_0_ is the volume at the beginning of the treatment. The therapeutic effect of the compounds was expressed as the volume ratio of treatment to control (T/C): T/C (%) = 100% ×(mean RTV of the treated group / mean RTV of the control group).

### Detection of microvessels and pericytes expression in tumour tissue

Paraffin sections of tumour were deparaffinized and hydrated, and endogenous peroxidase activity was blocked with 3% H_2_O_2_. Antigen retrieval was performed with a microwave oven. The slides were blocked with blocking serum from the host of the secondary antibody and incubated with primary antibodies anti-CD31 1:100 (Santa Cruz Biotechnology) or anti- PDGFRβ1:50 (Cell Signaling Technology) at 4°C overnight. Biotinylated secondary antibodies were added at a 1:100 dilution, followed by Vectastin ABC solution 1:100. The binding of the antibodies was visualized with 3,3-diaminobenzidine solution. Slides were then dehydrated and counterstained with harris' hematoxylin. The tissue sections were observed at ×200 magnification and tissue image was captured with a digital camera. Four fields per section were randomly analysed, excluding peripheral surrounding connective tissues and central necrotic tissues.

## Results

### AL3810 inhibits multiple tyrosine kinases activities *in vitro*

The effect of AL3810 on tyrosine kinase activities was evaluated with enzyme-linked immunosorbent assay (ELISA), and sixteen different kinases were analysed. Sorafenib, a potent multiple tyrosine kinase inhibitor, was selected as a positive control. AL3810 inhibited the angiogenesis-regulating kinases VEGFR1, VEGFR2, FGFR1, PDGFRα and PDGFRβ in the sub-nanomolar concentration, with IC_50_ values of 12, 4, 7,13 and 8 nmol/l respectively, about 10-fold lower than that of sorafenib. In addition, AL3810 also significantly inhibited activity of RET, c-KIT, EPHA2, EPHB2 and c-Src, with IC_50_ of 0.2, 0.527, 6.66, 0.902 and 4.9 μmol/l, identical to that of sorafenib, but had little effect on other tested tyrosine kianses, including EGFR, ErbB2, ABL, C-MET, IGF1R and IR (Fig. 1B).

### AL3810 functions as a reversible, ATP competitive tyrosine kinase inhibitor

**Fig 2:**
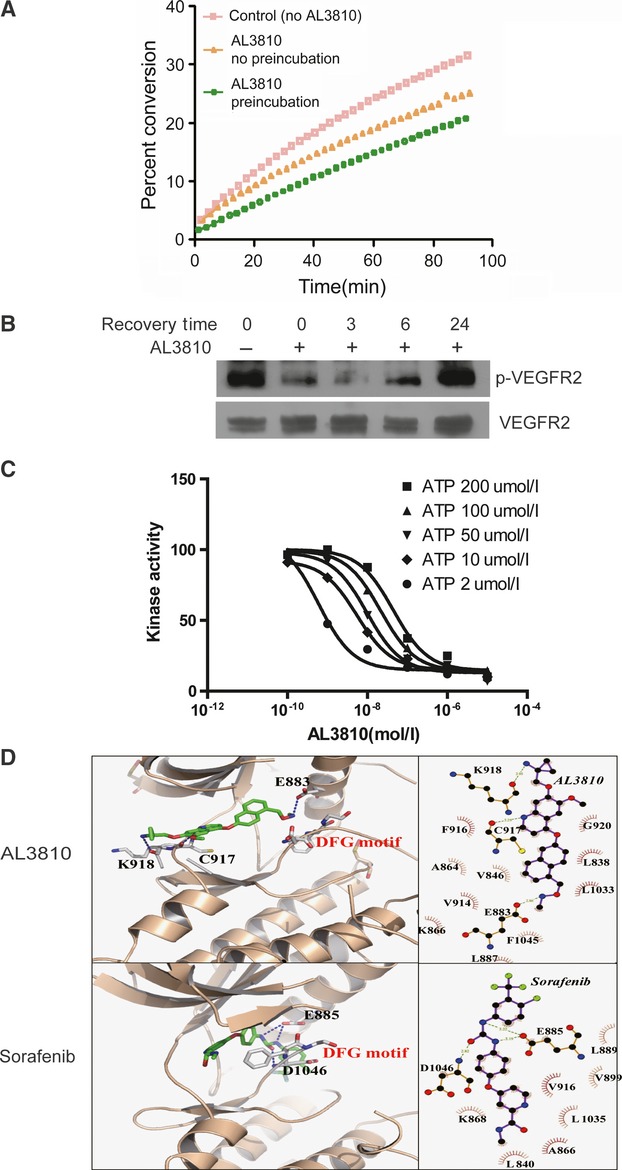
AL3810 functions as a reversible, ATP competitive tyrosine kinase inhibitor. (A) Using Caliper EZ Reader to assay the enzyme activity of VEGFR2 under three different conditions: without AL3810, with AL3810 and pre-incubated with AL3810, and the enzyme activity was marked by the percent of converge of substrate peptide (5-FAM-EEPLYWSFPAKKK-CONH2) (B) The inhibitory effect of AL3810 on VEGFR2 autophosphorylation in HUVEC cells is reversible. HUVEC cells were treated with AL3810 1 μM for 4 hrs, then AL310 was washed off and cells were cultured with fresh medium continuatively, at different time points (0, 3, 6, 24 hrs), cells were lysed and the lysate was subjected to SDS-PAGE to detect the phosphorylation of VEGFR2. (C) VEGFR2 kinase assays were performed as described in Materials and methods in the presence of varying concentrations of ATP. (D) The binding mode analysis of AL3810 with the active state of VEGFR2 and sorafenib with the inactive state of VEGFR2 respectively. DFG motif was shown in grey. All interactions shown in right panel were calculated by LIGPLOT program and the interactions shown in left panel were made by pymol software.

Kinase inhibitor is divided to irreversible and reversible inhibitors. To verify the intrinsic property of AL3810, a dilution method was used. VEGFR2 and AL3810 were incubated for 30 min. to allow the formation of an enzyme-inhibitor complex, and phosphorylated product was measured to indicate the enzymatic activity over time. The change in the rate of product formation reflects the dissociation of the enzyme-inhibitor complex. As shown in Figure 2A, after pre-incubation with AL3810, VEGFR2 activity recovered in a quick manner, suggesting that the compound is a reversible inhibitor of VEGFR2. To further clarify the binding profiles, the recovery of VEGFR2 phosphorylation after AL3810 washout was examined in HUVEC cells. Treatment with 1 μmol/L of AL3810 for 4 hrs caused a dramatic decrease in the level of phospho-VEGFR2. Upon the removal of the inhibitor, VEGFR2 phosphorylation was recovered substantially at 6 hrs, followed by a total restoration at 24 hrs after inhibitor washout (Fig. 2B), indicating that AL3810 is a reversible inhibitor. As most small molecule inhibitors are designed to block kinase activity *via* binding to the ATP pocket of the enzyme, we next used VEGFR2 as an example to investigate the binding mode of AL3810 with the enzyme. We found that the inhibitory effect of AL3810 on VEGFR2 kinase increased along with the increase of ATP concentration (Fig. 2C), suggesting that AL3810 is an ATP competitive tyrosine kinase inhibitor.

### AL3810 blocks cellular tyrosine kinase phosphorylation and downstream signalling pathways

**Fig 3:**
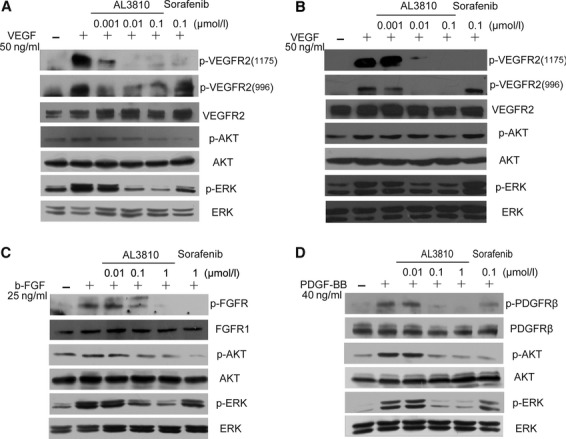
AL3810 blocks tyrosine kinase phosphorylation and downstream signalling. (A, B) AL3810 blocks VEGFR2 phosphorylation and its downstream signal transduction in HUVEC cells (A) and VEGFR2-NIH3T3 cells (B). (C) Inhibition of b-FGF-induced FGFR phosphorylaion and signal transduction by AL3810 in HUVEC cells. (D) Inhibition of PDGF-BB-induced PDGFRβ phosphorylation and signal transduction by AL3810 in Swiss3T3 cells. Cells were starved, then incubated with indicated concentrations of AL3810 for two hrs, VEGF_165_(50 ng/ml), b-FGF(25 ng/ml), PDGF-BB(40 ng/ml) was added to the cultures during the last 10 min. of the treatment.

We further elucidated the effect of AL3810 on targeted receptor tyrosine kinases using NIH-3T3 transfected with VEGFR2, HUVEC cells expressingVEGFR2 and FGFR1, as well as rat fibrolast Swiss 3T3 cells which express PDGFR. When NIH-3T3-VEGFR2 and HUVECs were incubated with AL3810 for 2 hrs followed by subsequent exposure to VEGF165, a concentration-dependent inhibition of VEGFR2 autophosphorylation was observed (Fig. 3A and B). AL3810 at 10 nmol/l almost completely blocked the autophosphorylation of VEGFR2. In addition, AL3810 dramatically inhibited VEGF-induced activation of ERK1/2 and AKT. Similarly, AL3810 blocked bFGF-induced autophosphorylation of FGFR1 and downstream signalling in HUVEC (Fig. 3C). Furthermore, AL3810 at 100 nmol/l also inhibited PDGFBB-induced PDGFRβ autophosphorylation in Swiss3T3 cell (Fig. 3D). Sorafenib also showed potent inhibitory effects on FGFR1 and PDGFRβ activation. Moreover, sorafenib exhibited a similar effect on Tyr1175 phosphorylation of VEGFR2, but little effect on Tyr996 phosphorylation (Fig. 3A and B).

### AL3810 is predicted to bind to the active form of VEGFR2

To acquire further insights into interaction mode of AL3810 with the tyrosine kinases, the automated docking stimulation exemplified by AL3810-VEGFR2 and Sorafenib-VEGFR2 interaction was performed. The crystal structure of human VEGFR2 was utilized as the target structure. We compared the binding modes of AL3810 with both active and inactive conformations of VEGFR2. We found that AL3810 docked well to the active conformation of VEGFR2, whereas sorafenib preferred to the inactive conformation of VEGFR2. In particular, AL3810 hydrogen bound to Glu883, Cys917 and Lys918 within the distances of 2.86, 3.29 and 2.95 Å, respectively (Fig. 2D). Further analysis revealed that AL3810 hydrophobically interacted with the active VEGFR2 with a series of aromatic rings and non-polar residues (Fig. 2D). Comparatively, sorafenib bound to E885 and D1046 of inactive VEGFR2 within the distance of 3.19, 3.22 and 2.82 Å, respectively, and hydrophobically interacted with the L840, A866, L889, V899, V916 and L1035 of VEGFR2 (right panel of Fig. 2D).

### AL3810 exhibits the potent anti-angiogenesis activity

**Fig 4:**
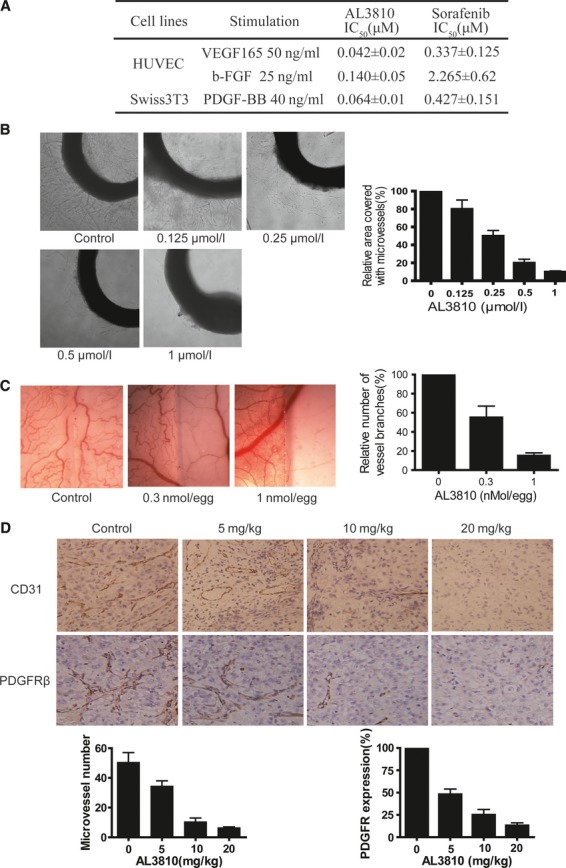
AL3810 has potent anti-angiogenesis activity. (A) The inhibitory activity of AL3810 on VEGF-, b-FGF- driven proliferation of HUVEC cells and PDGF-BB-driven proliferation of Swiss3T3 cells. Concentration that cause 50% inhibition (IC_50_) is shown as mean ± S.D. of three to six independent experiments performed. Cells proliferation was assayed as described in Materials and methods. (B) Effect of AL3810 on microvessel outgrowth arising from rat aortic rings. Aortic rings were embedded in Matrigel in 96-well plates, and then fed with medium containing various concentrations of AL3810 for 6 days. *Left*, representative photography (100×) of two independent experiments. *Right,* the area of microvessels was quantified and normalized to untreated controls. (C) Effects of AL3810 on angiogenesis in a chorioallantoic membrane model. Glasscover-slip saturated with AL3810 or normal saline was placed areas between pre-existing vessels in the fertilized chicken eggs and incubated for 48 hrs. The coverglass saturated with AL3810 was placed on the right side of per field. *Left*, representative photography (100×) of two independent experiments. *Right,* the number of vessel branches was quantified and normalized to untreated controls. (D) Nude mice with established renal carcinoma xenograft (No.213847) were treated with vehicle or AL3810 for five consecutive days at dosage of 5, 10, 20 mg/kg respectively, and then the tumour tissue were perfomed immunohistochemistry analysis by stained CD31 or PDGFRβ antibody. Four fields per section were captured and analysed. *Upper,* representative photography (200×) of three independent tumour tissues per group. *Below,* the micovessel number or expression of PDGFRβ per group was quantified.

Growth factors have been well defined as positive mediators of endothelial cell proliferation and angiogenesis. We then investigated whether AL3810 can block ligand-dependent cell proliferation. As shown in Figure 4A, AL3810 displayed potent inhibitory activity against VEGF-, bFGF-driven HUVECs proliferation and PDGFBB-driven Swiss3T3 proliferation (IC_50_: 0.042, 0.140 and 0.064 μmol/l respectively). The potency of AL3180 was around 10-fold stronger than that of sorafenib (IC_50_: 0.337, 2.265 and 0.427 μmol/l).

Next, we examined the effects of AL3810 on microvessel sprouting from vascular tissues using an *ex vivo* rat aortic ring angiogenesis assay, which mimics several stages in angiogenesis. Treatment with AL3810 resulted in a dramatic dose-dependent decrease in capillary sprouting; the growing sprouts were shorter with fewer cells migrating into the matrix. Moreover, we found that AL3810 caused a dose-dependent suppression in microvessel formation, with 51.5% inhibition observed at 0.25 μmol/l, and 90.5% inhibition at 1 μmol/l (Fig. 4B). Similar results were observed in CAM assay, another widely used *in vivo* model to evaluate angiogenesis. We found that AL3810 led to a dramatic and dose-dependent reduction in blood vessel numbers and branching patterns, with concentrations of 0.3 and 1 nmol/egg yielding inhibition rates of 45.4% and 82.5% respectively (Fig. 4C).

### AL3810 decreases both tumour microvessels and pericytes *in vivo*

To confirm the effect of AL3810 on tumour vasculature, mice with established renal carcinoma xenograft (No.213847) were treated with vehicle or AL3810 for five consecutive days at dosages of 5, 10, 20 mg/kg respectively. After the last administration, tissues were dissected and analysed by immunohistochemistry using CD31 and PDGFRβ antibodies to detect endothelial cells and pericytes,. In comparison with control group, vessel density in xenograft from mice treated with AL3810 dramatically decreased, with a reduction of 45%, 80%, 90% respectively (Fig. 4D). Quantification of PDGFRβ positive cells also showed a reduction of 52%, 73%, 86% respectively after 5 days of AL3810 administration (Fig. 4D). These results indicated that AL3810 counteracted tumour angiogenesis through simultaneously inhibiting vascular endothelial cells and pericytes, two important participants of vasculature.

### AL3810 inhibits tumour growth in a panel of human xenograft models

**Fig 5:**
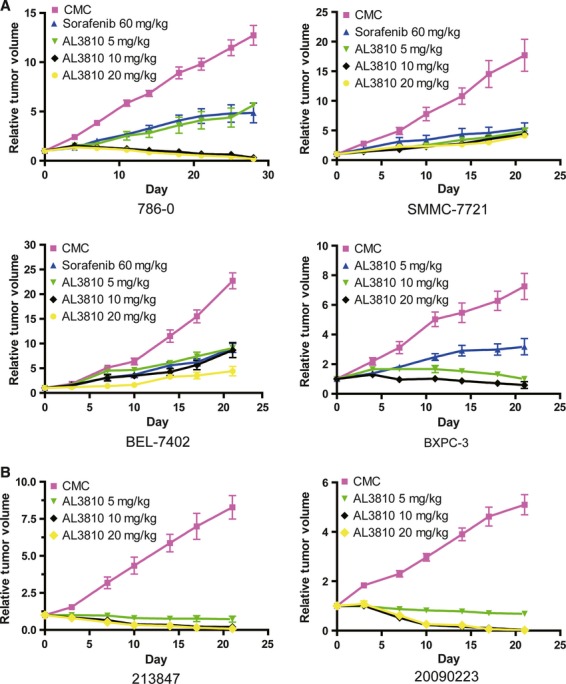
Anti-tumour activity of AL3810 *in vivo*. (A) Activity of AL3810 was determined in four human tumour xenograft including renal carcinoma 786-O, hepatocellular cancer SMMC-7721, BEL-7402, and pancreatic cancer BXPC-3 in nude mice. The animals were randomly divided to groups when tumour volume reached 100–200 mm^3^. In all models, the dosage of AL3810 is 5, 10, 20 mg/kg, p.o, 5 days per week. In models of 786-O, SMMC-7721, BEL-7402, the dosage of sorafenib is 60 mg/kg, p.o, 5 days per week. (B) The therapeutic effect of AL3810 on fresh tumour tissues from two patients with renal cell carcinoma (No.213847 and 20090223). The dosage of AL3810 is 5, 10, 20 mg/kg, p.o, 5 days per week. The value of RTV (relative tumour volume) = *V*_t_/*V*_0_, where *V*_t_ is the volume on each day, and *V*_0_ is the volume at the beginning of the treatment.

To further explore the anti-tumour efficacy of AL3810 *in vivo*, a panel of human xenograft models were used, including 786-O renal carcinoma, SMMC-7721, BEL-7402 hepatocellular cancer, and Bxpc-3 pancreatic cancer. Importantly, the p.o. administration of AL3810 for 5 days per week at dosage of 5–20 mg/kg caused a potent suppression of tumour growth with no obvious toxicity observed. At the dosage of 20 mg/kg, the T/C values were 1% (786-O model), 23% (SMMC-7721 model), 19% (BEL-7402 model) and 8% (Bxpc-3 model) respectively. Encouragingly, tumour regression was observed in 786-O and Bxpc-3 models, at dosages below 20 mg/kg (Fig. 5A).

To further evaluate the therapeutic efficacy of AL3810 in renal carcinoma, two renal carcinoma xenografts derived from patients (No.213847 and 20090223) were used (Fig. 5B). Similarly, AL3810 substantially inhibited the tumour growth with a T/C value of 9.01%, 13.3%, at 5 mg/kg respectively. And at the dose 10–20 mg/kg, AL3810 also caused tumour regression.

### AL3810 possesses a distinct pharmacokinetic profile

**Fig 6:**
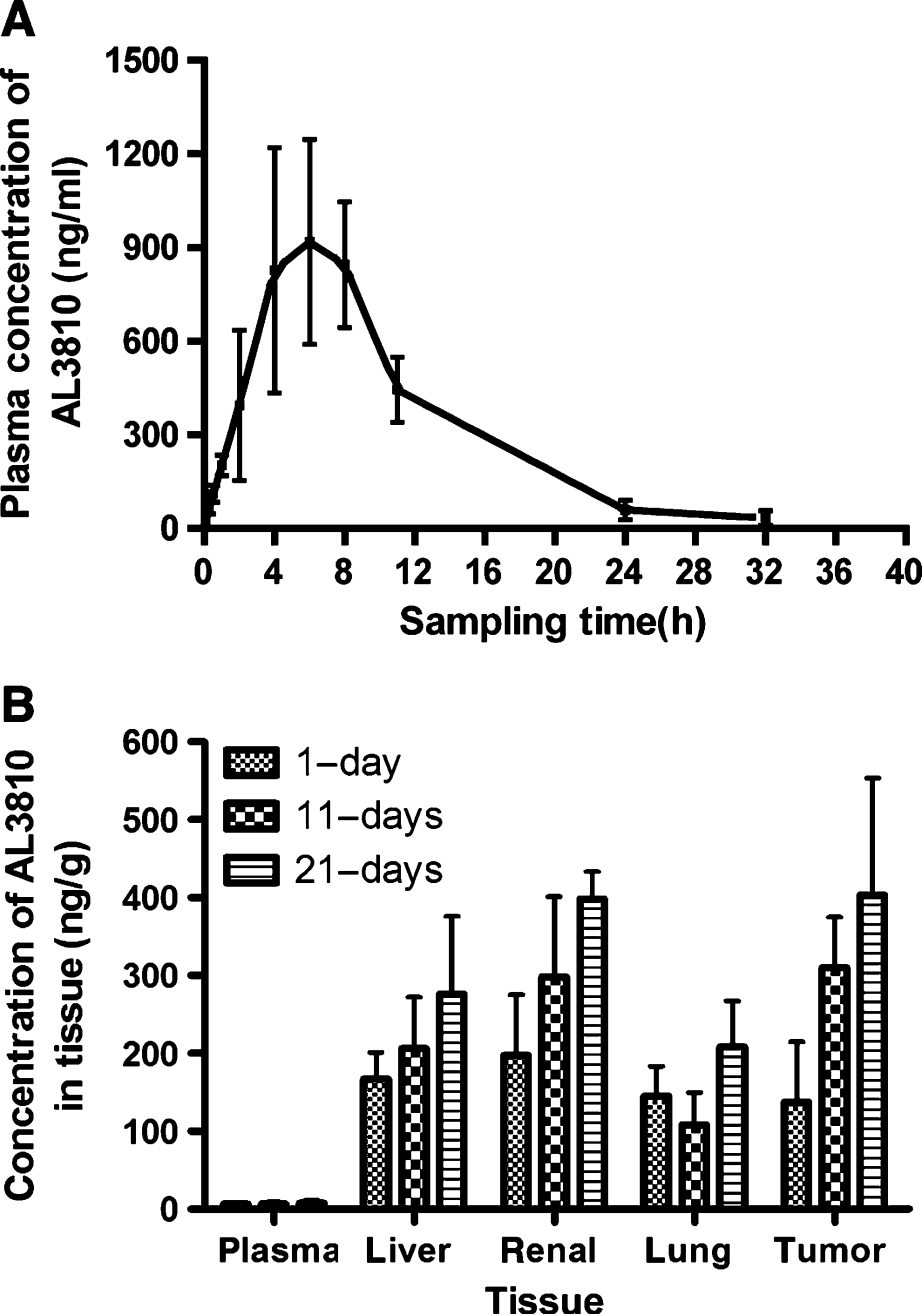
The pharmacokinetic profiles of AL3810. (A) AL3810 plasma concentration in mice after a single p.o. dose of 10 mg/kg (*n* = 8), (B) The concentration of AL3810 in different tissue of nude mice with established hepatocarcinoma (Bel-7402) after 1, 11, 21 day consecutive administration at dose of 10 mg/kg (*n* = 8).

Next, we characterized the pharmacokinetics profiles of AL3810. We found that 10 mg of AL3810 *via* p.o administration to rats revealed a maximal plasma concentration of ∼1000 ng/ml at 6 hrs and trough plasma level below 50 ng/ml at 24 hrs after administration (Fig. 6A). The oral bioavailability of AL3810 in rats was 31% (data not shown). The distribution of AL3810 in different tissues was studied in tumour-bearing nude mice. Our results revealed that the concentration of AL3810 in tissues was substantially higher than that in plasma. After a 21-day continuous administration, the concentrations of AL3810 in liver, renal, lung and tumoural tissues reached 276, 398, 208, 403 ng/g respectively, much higher than that in plasma (7.8 ng/ml), and in contrast to the first administration, the exposure of AL3810 in liver, renal, lung and tumours has improved 1.6, 2.1, 1.4 and 2.9 times respectively (Fig. 6B).

## Discussion

This report described the biochemical and pharmacological activity profile of AL3810, a low-nanomolar inhibitor of VEGFR, FGFR and PDGFR kinases with potential to inhibit the pro-angiogenic signalling pathways in vascular endothelial cells as well as pericytes, cell types that have attracted much attention as contributors to blood vessel formation, maturation and stabilization.

Thus far, a number of angiogenic inhibitors that target the VEGF/VEGFR signalling pathway have been validated to provide clinical benefit. However, a major problem noticed during the clinical development is the compensatory mechanisms for tumour angiogenesis that account for acquired resistance to VEGF-targeted therapy [29]. Of the complicated mechanisms, the engagement of FGF family is the major reason. It has been shown that treatment with an anti-VEGFR2 mAb was associated with a decrease in vascular density after 10 days of treatment. However, an angiogenic rebound in tumours at 4 weeks that was associated with an increase in expression of members of the FGF family [30]. An increase in the circulating levels of bFGF has been examined when tumours progressed on VEGF-targeted therapy [31]. Pericytes mediate blood flow and endothelial cell permeability, and direct contact between pericytes and endothelial cells enhances endothelial cell survival [32]. The proliferation and migration of pericytes is predominantly mediated by PDGF-BB (secreted primarily by endothelial cells) interacting with the PDGF receptor on pericytes. And a number of studies have shown that targeting both pericytes and endothelial cells (i.e. PDGF-R and VEGFR inhibitors) leads to greater efficacy than either agent alone [33, 34].

Given the complexity of tumour angiogenesis, simultaneously blocking FGF and PDGF signalling pathways during the inhibition of VEGF pathway may maximize the anti-tumour efficacy and minimize the acquired resistance to VEGF-targeted therapy. In the present study, we identified that AL3810 is a multi-angiogenic tyrosine kinases inhibitor significantly targeting VEGFR, FGFR and PDGFR kinases with a sub-nanomolar potency. These outstanding features endow AL3810 a broad inhibition of multiple pro-angiogenic signalling pathways in both vascular endothelial cells and pericytes both *in vitro* and *in vivo*. There is a discrepancy about PDGFR inhibition in contrast with the data reported by Bello *et al*. [17], they showed that this compound only had some effect on PDGFRα and PDGFRβ phosphorylation *in vitro* assay (IC_50_ values of 175 and 525nmol/L respectively) and in cell culture. In addition to the molecular assay, we also demonstrated that AL3810 could inhibit PDGF-BB driven Swiss3T3 cells proliferation (IC_50_ 0.076 μmol/l, Fig. 4A), and almost completely block PDGFR β phosphorylation in Swiss3T3 cell at 100 nmol/l (Fig. 3D). *In vivo* study showed the same result again. Nude mice with established renal carcinoma xenograft (No.213847) were treated with AL3810 for five consecutive days significantly reduced PDGFRβ positive cells in tumour tissue (Fig. 4D). All these data what we got, well supported the belief that AL3810 is also a potent PDGFR β inhibitor. We also have tested the inhibitory effect of AL3810 against 16 different tumour cell lines proliferation *in vitro* (in supplementary data), the average IC_50_ is about 18.7 μM, much higher than the IC_50_ of AL3810 on endothelial cell proliferation, which means that the anti-tumour potency of AL3810 mainly owe to its potent anti-angiogenic activity.

AL3810 was discovered using a combinatorial lead-optimization strategy. Inspite of the multiple targets selection, to gain the better biological activity, solubility and commercial availability of AL3810, a pyridine ring was fused with phenyl ring to introduce quinoline moiety to the structure, with the substitution of carbon moiety with primary amine. In addition, the urea was removed to simplify the molecule. Such chemical manipulations allow AL3810 a binding mode distinct from sorafenib. AL3810 bound in the region occupied by the adenine ring of ATP (the active conformation) [35], however, sorafenib bound the hydrophobic site that is adjacent to the ATP bind pocket (the inactive conformation). The structurally novel skeleton with subsequent distinct binding mode offers AL3810 appreciable biological functions. In the present study, we demonstrated that AL3810 exhibited stronger inhibitory potency than sorafenib in almost all tested models both *in vitro* and *in vivo*. In molecular assay, these two compounds showed similar potency against VEGFR2 and PDGFR, but AL3810 has higher potency against FGFR than sorafenib (IC_50_ values 25-fold lower than sorafenib). At cellular level, we demonstrated that 42–140 nM of AL3810 were required to achieve 50% cells growth inhibition against VEGF-, bFGF-driven HUVECs proliferation and PDGF-BB-driven Swiss3T3 proliferation, which was 10-folds lower than sorafenib. Importantly, AL3810 inhibited the phosphoryaltion on Tyr996 of VEGFR2, but sorafenib failed to exhibit such function. This may be because of the different interaction mode of these two molecular on VEGFR2, which was observed in the molecular modelling. AL3810 is predicted to bind to the kinase domain of VEGFR2, but Sorafenib may prefer to bind to the hydrophobic region besides to the kinase domain, in Figure 2D. Furthermore, in all *in vivo* xenograft models, the significant growth inhibition of AL3810 was achieved at doses of 10 mg/kg (daily p.o.) whereas the comparable anti-tumour efficacy of sorafenib, required six-fold higher dosage.

The s.c. xenograft tumours models derived from cell lines have been widely used to screen the anticancer drugs. However, recently a correct prediction of clinical outcome from xenografts derived from patient biopsied was observed for both tumour resistance (97%) and tumour sensitivity (92%) compared with clinical response [36]. The value of this kind of xenograft models for predicting clinical activity is recognized now. Therefore, in addition to the classical xenograft models, two renal carcinoma xenografts derived from patients were used to further help predict the clinical response to AL3810 in this study. Also, the glad thing is that the tumours were almost gone after treatment with 20 mg/kg AL3810 in these two patient derived xenograft models.

In summary, we have identified AL3810, an orally active inhibitor against multiple angiogenic tyrosine kinases that demonstrates significant anti-angiogenesis efficacy and anti-tumour activity *in vitro*, *ex vivo* and *in vivo*. These data, together with its superior pharmacokinetic profiles with good orally bioavailability and desirable tissue distribution, provide a strong rational for clinical trial of AL3810 which will soon be launched in China.

## Supporting information

**Figure S1** The inhibitory effect of AL3810 on tumour cells proliferation (mean IC_50_ were calculated from at least three independent experiments).

## References

[1] Bergers G, Benjamin LE (2003). Tumorigenesis and the angiogenic switch. Nat Rev Cancer.

[2] Folkman J (2007). Angiogenesis: an organizing principle for drug discovery?. Nat Rev Drug Discov.

[3] Kowanetz M, Ferrara N (2006). Vascular endothelial growth factor signaling pathways: therapeutic perspective. Clin Cancer Res.

[4] Keck PJ, Hauser SD, Krivi G (1989). Vascular permeability factor, an endothelial cell mitogen related to PDGF. Science.

[5] Lamoreaux WJ, Fitzqerald ME, Reiner A (1998). Vascular endothelial growth factor increases release of gelatinase A and decreases release of tissue inhibitor of metalloproteinases by microvascular endothelial cells *in vitro*. Microvasc Res.

[6] Lamoreaux WJ, Fitzgerald ME, Reiner A (1996). Angiogenesis: a paradigm for balanced extracellular proteolysis during cell migration and morphogenesis. Enzyme Protein.

[7] Shibuya M, Claesson-Welsh L (2006). Signal transduction by VEGF receptor in regulation of angiogenesis and lymphangiogenesis. Exp Cell Res.

[8] Andrae J, Gallini R, Betsholtz C (2008). Role of platelet-derived growth factors in physiology and medicine. Genes Dev.

[9] Jain RK (2003). Molecular regulation of vessel maturation. Nat Med.

[10] Benjamin LE, Hemo I, Keshet E (1998). A plasticity window for blood vessel remodelling is defined by pericyte coverage of the preformed endothelial network and is regulated by PDGF-B and VEGF. Development.

[11] Erber R, Thurnher A, Katsen AD (2004). Combined inhibition of VEGF and PDGF signaling enforces tumour vessel regression by interfering with pericyte-mediated endothelial cell survival mechanisms. FASEB J.

[12] Presta M, Dell'Era P, Mitola S (2005). Fibroblast growth factor/fibroblast growth factor receptor system in angiogenesis. Cytokine Growth Factor Rev.

[13] Seghezzi G, Patel S, Ren CJ (1998). Fibroblast growth factor-2 (FGF-2) induces vascular endothelial growth factor (VEGF) expression in the endothelial cells of forming capillaries: an autocrine mechanism contributing to angiogenesis. J Cell Biol.

[14] Tsunoda S, Nakamuia T, Sakurai H (2007). Fibrolast growth factor-2-induced host stroma reaction during initial tumour growth promotes progression of mouse melanoma *via* vascular endothelial growth factor A-dependent neovascularization. Cancer Sci.

[15] Gotink KJ, Verheul HM (2010). Anti-angiogenic tyrosine kinase inhibitors: what is their mechanism of action?. Angiogenesis.

[16] Cao H, Zhang H, Zheng X (2007). 3D QSAR studies on a series of potent and high selective inhibitors for three kinases of RTK family. J Mol Graph Model.

[17] Bello E, Colella G, Scarlato V (2011). E-3810 is a potent dual inhibitor of VEGFR and FGFR that exerts antitumour activity in multiple preclinical models. Cancer Res.

[18] Wilhelm SM, Carter C, Tang L (2004). BAY 43-9006 exhibits broad spectrum oral antitumour activity and targets the RAF/MEK/ERK pathway and receptor tyrosine kinases involved in tumour progression and angiogenesis. Cancer Res.

[19] Jaffe EA, Nachman RL, Becker CG (1973). Culture of human endothelial cells derived from umbilical veins. Identification by morphologic and immunologic criteria. J Clin Inves.

[20] Zhang YX, Chen Y, Guo XN (2005). 11,11′-dideoxy-verticillin: a natural compound possessing growth factor receptor tyrosine kinase-inhibitory effect with anti-tumour activity. Anticancer Drugs.

[21] Guo XN, Zhong L, Zhang XH (2004). Evaluation of active recombinant catalytic domain of human ErbB-2 tyrosine kinase, and suppression of activity by a naturally derived inhibitor, ZH-4B. Biochim Biophys Acta.

[22] Bernstein FC, Koetzle TF, Williams GJ (1977). The Protein Data Bank: a computer-based archival file for macromolecular structures. J Mol Biol.

[23] Friesner RA, Banks JL, Murphy RB (2004). Glide: a new approach for rapid, accurate docking and scoring. 1. Method and assessment of docking accuracy. J Med Chem.

[24] Wallace AC, Laskowski RA, Thornton JM (1995). LIGPLOT: a program to generate schematic diagrams of protein-ligand interactions. Protein Eng.

[25] DeLano WL (2002). The pymol user's manual.

[26] Poirel L, Laqrutta E, Taylor P (2010). Emergence of metallo-ss-lactamase NDM-1-producing multidrug resistant *Escherichia coli* in Australia. Antimicrob Agents Chemother.

[27] Yang X, Luo P, Yang B (2006). Antiangiogenesis response of endothelial cells to the antitumour drug 10-methoxy-9-nitrocamptothecin. Pharmacol Res.

[28] Staton CA, Stribbling SM, Tazzyman S (2004). Current methods for assaying angiogenesis *in vitro* and *in vivo*. Int J Exp Pathol.

[29] Broxterman HJ, Gotink KJ, Verheul HM (2009). Understanding the causes of multidrug resistance in cancer: a comparison of doxorubicin and sunitinib. Drug Resist Updat.

[30] Casanovas O, Hicklin DJ, Bergers G (2005). Drug resistance by evasion of antiangiogenic targeting of VEGF signaling in late-stage pancreatic islet tumours. Cancer Cell.

[31] Batchelor TT, Sorensen AG, di Tomaso E (2007). AZD2171, a pan-VEGF receptor tyrosine kinase inhibitor, normalizes tumour vasculature and alleviates edema in glioblastoma patients. Cancer Cell.

[32] Raza A, Franklin MJ, Dudek AZ (2010). Pericytes and vessel maturation during tumour angiogenesis and metastasis. Am J Hematol.

[33] Klosowska-Wardega A, Hasumi Y, Burmakin A (2009). Combined anti-angiogenic therapy targeting PDGF and VEGF receptors lowers the interstitial fluid pressure in a murine experimental carcinoma. PLoS One.

[34] Kuhnert F, Tam BY, Sennino B (2008). Soluble receptor-mediated selective inhibition of VEGFR and PDGFRbeta signaling during physiologic and tumour angiogenesis. Proc Natl Acad Sci.

[35] Liu Y, Gray NS (2006). Rational design of inhibitors that bind to inactive kinase conformations. Nat Chem Biol.

[36] Fiebig HH, Schuchhardt C, Henss H (1984). Comparison of tumour response in nude mice and in the patients. Behring Inst Mitt.

