# Supplementary figures and images for: AL3810, a multi-tyrosine kinase inhibitor, exhibits potent anti-angiogenic and anti-tumour activity via targeting VEGFR, FGFR and PDGFR
Source: J Cell Mol Med. 2012 Sep 26;16(10):2321–30. doi: 10.1111/j.1582-4934.2012.01541.x (PMC3823425; doi:10.1111/j.1582-4934.2012.01541.x)

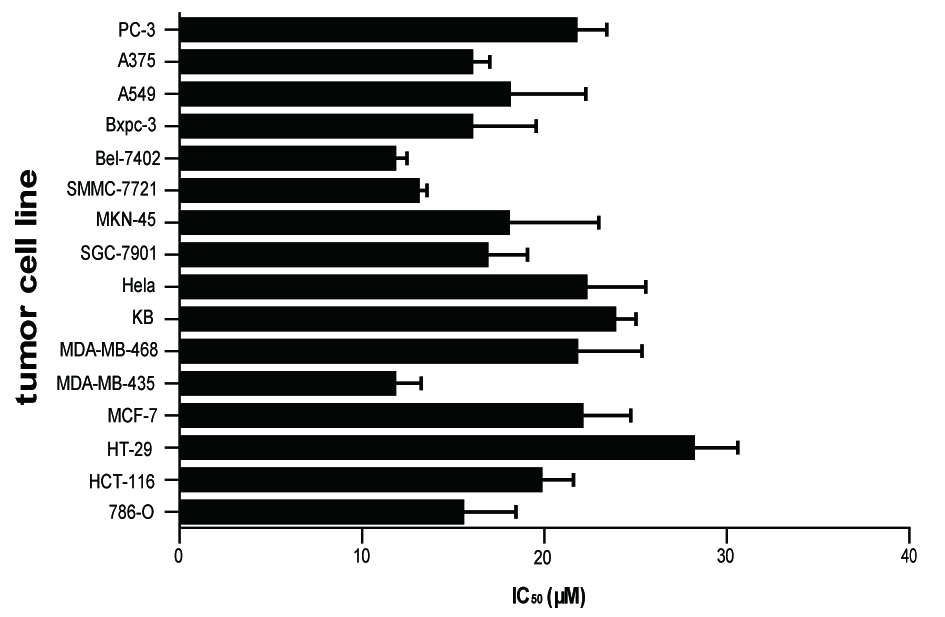

Supplement: Supplementary file 1 [file jcmm0016-2321-SD1.tif]
